# Retrospective Analysis of Bevacizumab in Combination with Fotemustine in Chinese Patients with Recurrent Glioblastoma Multiforme

**DOI:** 10.1155/2015/723612

**Published:** 2015-02-18

**Authors:** Zhiguang Liu, Guanqun Zhang, Liang Zhu, Jiangbo Wang, Dongbo Liu, Lifei Lian, Jianlin Liu, Tianbao Lai, Xiaorong Zhuang

**Affiliations:** ^1^Department of Neurology, Xuzhou Center Hospital, Xuzhou 221009, China; ^2^Department of Cancer, Tongji Hospital, Tongji Medical College, Huazhong University of Science and Technology, Wuhan 430030, China; ^3^Department of Neurology, Tongji Hospital, Tongji Medical College, Huazhong University of Science and Technology, Wuhan 430030, China; ^4^Department of Neurology, Zhongshan Hospital, Xiamen University, Xiamen 361004, China

## Abstract

The aim of this study was to assess the activity and safety of bevacizumab (BEV) and fotemustine (FTM) for the treatment of recurrent glioblastoma multiforme (GBM) patients and explore the potential prognostic parameters on survival. This study retrospectively analyzed all patients with GBM who were treated with at least one cycle of BEV and FTM from July 2010 to October 2012. A total of 176 patients with recurrent GBM were enrolled. The response rate and disease control rate were 46.6% and 90.9%, respectively. A 6-month PFS rate of 33.3% (95% CI: 26.5%–40.3%) and a median PFS of 5.0 (95% CI: 2.4–7.5) months were observed. The median OS was 8.0 (95% CI: 6.7–9.2) months. Multivariate analysis showed that risk factors with a significant influence on the PFS of all patients were Karnofsky Performance Status (KPS) (≥70 versus <70, HR = 0.53, 95% CI: 0.39–0.73, and *P* = 0.01) and MGMT status (methylated versus unmethylated, HR = 0.69, 95% CI: 0.52–0.97, and *P* = 0.04). The most common treatment-related adverse events were fatigue, proteinuria, hypophonia, hypertension, thrombocytopenia, anemia, and neutropenia. In conclusion, combination of BEV with FTM is well tolerated and may derive some clinical benefits in recurrent GBM patients. Higher KPS and MGMT promoter hypermethylation were suggested to be associated with prolonged survival.

## 1. Introduction

Glioblastoma multiforme (GBM) is the most common malignant brain tumor in adults and accounts for 17% of intracranial tumors [1]. The incidence in Europe and North America is 2-3 cases per 100,000 people annually [2]. Its aggressive clinical behavior leads to a dismal prognosis. The standard therapy involves maximal safe surgical resection, followed by radiotherapy with concomitant and adjuvant temozolomide (TMZ) [3, 4]. However, despite advances in the last 3 decades, long-term survival is rarely achieved and the disease almost invariably recurs. With disease recurrence, the available options are limited.

It is known that GBM is one of the most vascularized human tumors and that GBM cells produce proangiogenic factors, including VEGF. VEGF binds with its corresponding tyrosine kinase receptors, activating a downstream signal that results in the development of angiogenesis, increased vascular permeability, and lymphangiogenesis. Bevacizumab (BEV), a humanized monoclonal antibody against VEGF, alone or associated with chemotherapy or targeted medicines, has reported higher response rates and prolongation of median and 6-month progression-free survival compared to controls with non-bevacizumab treatments [5–7]. It was granted accelerated approval by the United States Food and Drug Administration (FDA) as an agent for the treatment of recurrent GBM. Nevertheless, the European Medicines Agency (EMA) refused this indication due to a lack of evidence. Nitrosoureas are in Europe the standard salvage option in recurrent GBM. The third-generation nitrosourea derivative fotemustine (FTM) is a cytotoxic alkylating agent showing activity in melanoma [8]. In a randomized trial comparing FTM with dacarbazine, as first-line chemotherapy in 229 patients, overall response rate in the intent-to-treat population was, respectively, 15.5% versus 6.8% [9]. Interestingly, among patients without brain metastases at inclusion, median time to development of central nervous system relapses was 22.7 months in the FTM arm versus 7.2 months in the group receiving dacarbazine. BEV and FTM have been shown to cross the blood-brain barrier (BBB) and demonstrated a favorable safety profile [10, 11].

The aim of this retrospective study was to evaluate the activity and safety of BEV and FTM for the treatment of recurrent GBM patients and explore the potential prognostic parameters on survival.

## 2. Materials and Methods

### 2.1. Patients

This study was a monocentric retrospective research conducted at Department of Neurology, Zhongshan Hospital, Xiamen University. All patients gave their written informed consent after being informed of the purpose and investigational nature of the study. The principles of the World Medical Association Declaration of Helsinki and Good Clinical Practice Guidelines were strictly followed.

Inclusion criteria for the study were as follows: (1) age ≥ 18 years; (2) histological diagnosis of glioblastoma at original surgery or at reoperation; (3) first progression after radiotherapy and concomitant/adjuvant temozolomide; (4) adequate hematological, liver, and renal function: hematocrit > 29%; absolute neutrophil count (ANC) ≥ 1,000 *μ*L; platelets count ≥ 100,000 *μ*L. Patients were excluded as follows: (1) evidence of hemorrhage on the baseline MRI or previous treatment including either BEV or FTM; (2) pregnancy or nursing; (3) uncontrolled hypertension; (4) cardiac arrhythmias; (5) history of congestive heart failure or stroke; (6) active infection requiring intravenous antibiotics; (7) urine protein : creatinine ratio > 1; (8) psychiatric disorders; (9) other conditions that would make the treatment unsafe.

### 2.2. Treatment

Eligible patients were treated with a combination of BEV and FTM. The treatment consisted of an induction phase with BEV at 10 mg/kg intravenously on days 1 and 15 and FTM at 75 mg/m^2^ intravenously on days 1 and day 8, followed after an interval of 3 weeks by a maintenance phase with BEV at 10 mg/kg and FTM at 75 mg/m^2^ every 3 weeks until tumor progression, withdrawal of the patient, or unacceptable toxicity. Ten minutes before each infusion of FTM, a 5-HT3 receptor antagonist was administered for antiemetic prophylaxis. Antiepileptic drugs were given during the study period as medically indicated. Glucocorticoids were used to the dose necessary for neurologic stability. FTM administration was held in case of grade 3-4 thrombocytopenia and/or neutropenia and grade 3-4 nonhematological toxicity except for nausea/vomiting. At recovery, treatment was resumed with a 25% dose reduction. BEV was discontinued for uncontrollable hypertension, grade 2 or greater hemorrhage, arterial thrombosis, severe proteinuria, or congestive heart failure. BEV was held until other related grade 3 events resolved to grade ≤1.

### 2.3. Clinical Evaluation

A full medical history was determined at treatment initiation and patients were examined for physical and neurological status, vital signs, and Karnofsky Performance Status (KPS) every 4 weeks. MRI scans were undertaken to evaluate tumor size and location every 8 weeks.

Tumor response was evaluated according to the MacDonald criteria every two treatment cycles or onset of progressive disease, considering MRI measurements of contrast-enhancing tumor size and recording of the largest cross-sectional area of the tumor and patient neurological status. Tumor response was codified as complete response, partial response, stable disease, and progressive disease when compared to best overall response scan. Progression-free survival (PFS) and overall survival (OS) were also recorded. PFS was defined as the time from the first administration of BEV and FTM to documented progression or death. OS was defined as the time from the first administration of BEV and FTM to date of death from any cause. Adverse events were graded according to NCI-CTCAE, version 3.0, criteria.

### 2.4. MGMT Gene Promoter Methylation Analysis

Genomic DNA was isolated from one paraffin section of glioblastoma tissue, denaturated with sodium hydroxide, and subjected to bisulfite treatment in a volume of 350 *μ*L (4.4 M sodium bisulfite and 20 mM hydroquinone) for five hours at 55°C and then purified. The methylation-specific PCR was performed in a two-step approach.

### 2.5. Statistical Analysis

All categorical variables are summarized and expressed as proportions. OS and PFS were estimated using the Kaplan-Meier method and the 95% confidence interval (95% CI) for median survival was estimated by the Brookmeyer-Crowley method. Moreover, subset analyses were conducted, using the Cox proportional hazard model, to identify factors that influenced OS and PFS. Statistical significance was defined as *P* values <0.05. All statistical analyses were performed with the SPSS statistical software program package (SPSS version 17.0 for windows, SPSS Inc., Chicago, Illinois, USA).

## 3. Results

### 3.1. Patient Characteristics

A total of 176 consecutive patients, including 111 men and 65 women, with recurrent GBM admitted to Department of Neurology, Zhongshan Hospital, Xiamen University, between July 2010 and October 2012, were enrolled. The patient characteristics are summarized in Table 1. Median age was 56.5 years (range, 22–74 years). KPS (range 50–90) was ≥70 in 98 (55.7%) patients and <70 in 78 (44.3%) patients. 71.0% of patients were on steroids. 72 patients (40.9%) had MGMT unmethylated tumors while 65 (36.9%) had MGMT methylated tumors; in the remaining 39 patients data were not available. The median time from original diagnosis to study enrollment was 12 months. All patients completed the induction phase, and as a median 6 maintenance cycles were administrated (range 1–24).

### 3.2. Response and Survival Analysis

All follow-up assessments were completed by December 2013. Outcomes could be confirmed in all patients; no patient was lost to follow-up. At the end of the study, 2 patients were free of tumor progression and alive. The median follow-up for all patients was 9.5 months (95% CI 7.5–10.5).

All patients were evaluable for response rate. 3 cases of complete response were observed; 79 patients achieved partial response while 78 patients had disease stabilization, for a response rate of 46.6% and a disease control rate of 90.9%. The disease progressed in 16 patients.

**Figure 1:**
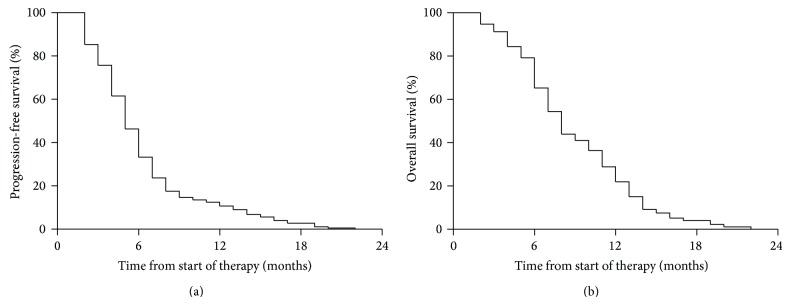
Kaplan-Meier survival curves of progressive-free survival and overall survival.

Median OS was 8.0 (95% CI: 6.7–9.2) months. At 6 and 12 months, 65.3% (95% CI: 57.7%–71.9%) and 22.0% (95% CI: 16.1%–28.4%) of patients were alive, respectively. OS differed with regard to response: 9.0 (95% CI: 8.3–9.9) months for responders and 6.5 (95% CI: 5.5–7.3) months for nonresponders (*P* < 0.01). PFS rates at 6 months and 12 months were 33.3% (95% CI: 26.5%–40.3%) and 10.7% (95% CI: 6.7%–15.8%) (Figure 1(a)). Median PFS was 5.0 (95% CI: 2.4–7.5) months and also differed with regard to response: 6.5 (95% CI: 5.3–7.7) months for responders and 3.0 (95% CI: 2.5–3.6) months for nonresponders (*P* < 0.01).

### 3.3. Prognostic Factors

**Figure 2:**
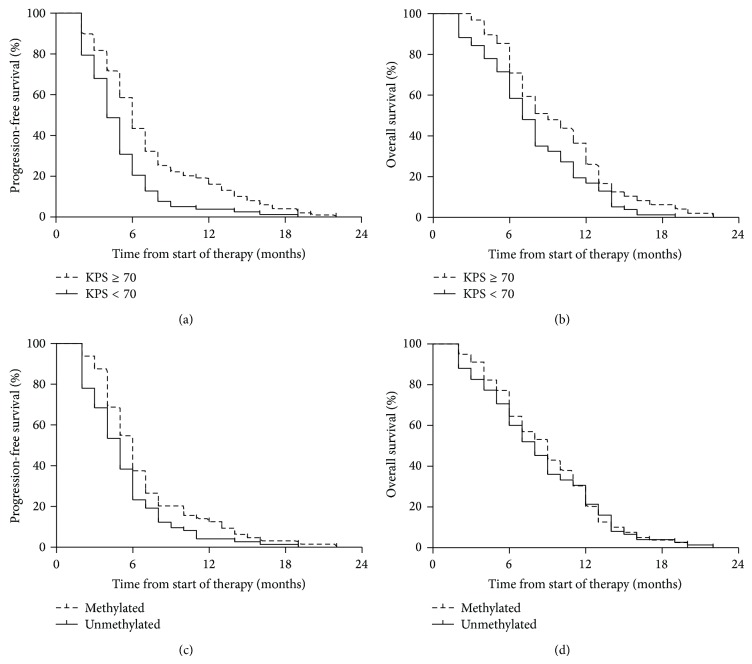
Kaplan-Meier survival curves of progressive-free survival and overall survival categorized according to the patients' KPS and MGMT status. Differences were analyzed with the log-rank test. Progressive-free survival was significantly higher (*P* < 0.01) in KPS ≥ 70 patients (a) and methylated patients (c), compared with KPS < 70 patients and unmethylated patients. Overall survival was significantly higher (*P* < 0.01) in KPS ≥ 70 patients (b), compared with KPS < 70 patients. The difference in the overall survival between methylated and unmethylated patients was not significant (d).

All potential prognostic variables were analyzed to establish their effect on progression-free and overall survival rates.  Table 2 showed that risk factors with a significant influence on the PFS of all patients were KPS (≥70 versus <70, HR = 0.49, 95% CI: 0.34–0.70, and *P* < 0.01) and MGMT status (methylated versus unmethylated, HR = 0.65, 95% CI: 0.44–0.95, and *P* = 0.03) (Table 2). Univariate analysis revealed that the median PFS for patients with KPS ≥ 70 was 6.0 (95% CI: 5.2–6.7) months, while it was 4.0 (95% CI: 3.4–4.7) months for patients with KPS < 70 (Figure 2(a)). The median PFS was 6.0 (95% CI: 4.9–6.9) months for methylated patients and 5.0 (95% CI: 4.1–5.8) months for unmethylated patients (Figure 2(c)). On multivariate stepwise regression hazards model analysis of prognostic factors, KPS (≥70 versus <70, HR = 0.53, 95% CI: 0.39–0.73, and *P* = 0.01) and MGMT status (methylated versus unmethylated, HR = 0.69, 95% CI: 0.52–0.97, and *P* = 0.04) were prognostic factors for PFS.

Both univariate analysis and multivariate analysis showed that KPS (univariate analysis, ≥70 versus <70, HR = 0.63, 95% CI: 0.45–0.89, and *P* = 0.01; multivariate analysis, ≥70 versus <70, HR = 0.58, 95% CI: 0.39–0.76, and *P* = 0.01) was the only prognostic factor to significantly impact OS (Table 3). The median OS for patients with KPS ≥ 70 was 9.0 (95% CI: 7.6–10.1) months, while it was 7.0 (95% CI: 6.1–8.1) months for patients with KPS < 70 (Figure 2(b)).

Gender, age, steroid use, type of first surgery, time from original diagnosis, second surgery, and maximum tumor diameter did not influence PFS and OS in both univariate and multivariate analysis (Tables 2 and 3).

### 3.4. Profile of Safety and Adverse Events

Haematologic and nonhaematologic adverse events are shown in Table 4. The most common treatment-related adverse events were fatigue, proteinuria, hypophonia, hypertension, thrombocytopenia, anemia, and neutropenia. Grade 3 toxicities were predominately hematologic, including neutropenia in 18 patients (10.2%), thrombocytopenia in 14 patients (8.0%), and anemia in 2 patients (1.1%). Other grade 3 toxicities included fatigue in 5 patients (2.8%), hypertension in 4 patients (2.3%), DVT in 3 patients (1.7%), and stroke, PE, and wound dehiscence in 1 patient (0.6%). Grade 4 toxicities included stoke and neutropenia in 3 patients (1.7%) and thrombocytopenia in 2 patients (1.1%). There were no treatment-related deaths in this study.

## 4. Discussion

Recently, several prospective and retrospective studies provided clinical data on BEV activity both as single agent and in combination therapy, establishing this antiangiogenetic agent as a valuable and active treatment option in GBM [12–14]. The choice of FTM, as the chemotherapy agent combined with BEV, was based on the rational that BEV might enhance the delivery of an active cytotoxic drug, and adequate safety would be expected with this regimen due to nonoverlapping primary toxicities of each of the agents [15]. This present retrospective analysis evaluated the combination of BEV and FTM in patients with recurrent GBM and explored the prognostic factors that might influence PFS and OS.

Our finding showed that the association of BEV with FTM achieved a response rate of 46.5% and a disease control rate of 90.9%. Similar results were reported by Soffietti et al. with a higher response rate (52%) and a lower disease control rate (89%) [15]. In our study, we observed a median OS of 8.0 months and a median PFS of 5.0 months. A series of clinical trials have evaluated the association of BEV with other miscellaneous agents, including irinotecan [16], etoposide [17], temozolomide [18], carboplatin [19], cetuximab [20], and erlotinib [21] in patients with recurrent GBM, showing median OS from 4 to 11.5 months and median PFS from 2 to 7.6 months. Based on these and the fact that the examined patients were consecutive and not selected, we conclude that the patients included are good representatives for the general population affected with GBM.

A difference in survival among patients with GBM has been found to depend on KPS and MGMT status. Maintenance of functional independence (KPS ≥ 70) is supposed as important component of clinical activity and treatment compliance for patients with a brain tumor, particularly those with recurrent GBM [13, 22]. In our study, univariate analysis indicated that both the median PFS and OS were higher for patients with KPS ≥ 70, compared with those with KPS < 70. Moreover, on multivariate analysis, KPS presented a HR of 0.53 for PFS and 0.58 for OS, suggesting that lower KPS affected survival adversely. These results are consistent with those of previous studies [23–25]. The prognostic significance of MGMT promoter methylation on GBM remains controversial. Some studies found longer PFS and OS in patients with MGMT promoter methylated tumors [26–28], whereas others did not [29–31]. A meta-analysis of published studies showed that MGMT promoter methylation was associated with better PFS (HR = 0.51; 95% CI: 0.38–0.69) and OS (HR = 0.49; 95% CI: 0.38–0.64) in patients with GBM regardless of therapeutic intervention and associated with longer OS (HR = 0.42; 95% CI: 0.29–0.60) in GBM patients treated with alkylating agents [32]. In our study we found an association between MGMT promoter methylation and longer PFS that was statistically significant in both univariate and multivariate analysis. Mounting evidence suggested a survival benefit in patients receiving a reoperation at the time of high-grade glioma recurrence [33]; however, in the present study second surgery was not determined to be an independent prognostic factor. It might be due to the small sample size of the study; in particular only 32 (18.2%) patients received the second surgery. Other prognostic markers for glioblastoma (e.g., gender, age, steroid use, type of first surgery, time from original diagnosis, and maximum tumor diameter) were also not predictive of survival, findings that were consistent with those in previous studies [22, 34].

The combination of BEV and FTM used herein is well tolerated. Most frequent grade 3-4 toxicities were primarily related to chemotherapy. BEV-related toxicities were consistent with those reported in other trials [10, 35]. In our research, 10.2% and 8.0% of patients suffered from grade 3 neutropenia and thrombocytopenia, respectively, while other grade 3 adverse effects (e.g. fatigue, hypertension, and DVT) were reported in a low percentage of patients. Only a few cases were reported to experience grade 4 toxicities. The safety of combination of BEV and FTM was also demonstrated by Soffietti et al.'s study. These outcomes also showed most patients experienced grade 1 or 2 toxicities and only a few patients experienced grade 4 or above toxicities.

Preliminary results of the Dutch phase II (BELOB trial), randomized trial on firstly recurrent GBMs comparing BEV plus lomustine versus either lomustine or BEV alone, showed a better outcome in terms of 6-month PFS and OS for the combination (single-agent bevacizumab or lomustine versus a combination of bevacizumab and lomustine in patients with recurrent glioblastoma (BELOB trial): a randomised controlled phase 2 trial). However, Soffietti et al.'s outcomes were unable to demonstrate a superiority of the combination of BEV and FTM over either BEV or FTM alone as historical control. Future studies should investigate whether a clinical benefit from the combination could emerge by employing different doses and schedules of BEV and FTM.

It should be noted that our study has some limitations. Even though Macdonald criteria were widely used to assess response to treatment of glioblastoma since published in 1990, they have been largely superseded by the RANO criteria which have become the mainstay of assessment (Monitoring Radiographic Brain Tumor Progression). Compared with RANO, the use of Macdonald criteria might yield a longer PFS because of later identification of progression. Furthermore, the sample size was small, and the physician that evaluated the MRI scans was not blind to clinical data.

## 5. Conclusions

According to the findings of the present study and review of the literature, GBM is a highly aggressive tumor which tends to have early relapse and short-term survival. This study shows that the combination of BEV with FTM is well tolerated and may derive some clinical benefits in recurrent GBM patients. Higher KPS and MGMT promoter hypermethylation were suggested to be associated with prolonged survival.

## Figures and Tables

**Table 1:** Patient characteristics.

Characteristics	Population (*n* = 176)
Gender, n (%)	
Female	65 (36.9)
Male	111 (63.1)
Median age, years (range)	56.5 (22–74)
KPS score, n (%)	
≥70	98 (55.7)
<70	78 (44.3)
Steroid use, n (%)	
No	51 (29.0)
Yes	125 (71.0)
Type of first surgery, n (%)	
Biopsy	17 (9.7)
Partial resection	66 (37.5)
Subtotal/total resection	93 (52.8)
MGMT status	
Unmethylated	72 (40.9)
Methylated	65 (36.9)
Not evaluable	39 (22.2)
Time from original diagnosis (months)	12 (3–122)
Second surgery before treatment	
No	144 (81.8)
Yes	32 (18.2)
Initial tumor size (mm^2^)	1800 (200–5300)
Initial maximum diameter (mm)	60 (15–105)

**Table 2:** Univariate and multivariate analysis of prognostic factors for progression-free survival in 176 patients with recurrent glioblastoma multiforme.

	Univariate effect			Multivariate effect		
	HR	95% CI	*P*	HR	95% CI	*P*
Gender						
Female	1			1		
Male	0.87	(0.62–1.43)	0.76	1.24	(0.78–1.67)	0.36
Age (years)	0.95	(0.85–1.12)	0.85	0.98	(0.91–1.25)	0.92
KPS score, *n* (%)						
<70	1			1		
≥70	0.49	(0.34–0.70)	<0.01	0.53	(0.39–0.73)	0.01
Steroid use						
No	1					
Yes	0.84	(0.63–1.38)	0.81	0.79	(0.57–1.19)	0.74
Type of first surgery, *n* (%)						
Biopsy/partial resection	1			1		
Subtotal/total resection	0.86	(0.49–1.42)	0.74	0.95	(0.52–1.54)	0.89
MGMT status						
Unmethylated	1			1		
Methylated	0.65	(0.44–0.95)	0.03	0.69	(0.52–0.97)	0.04
Time from original diagnosis (months)						
<12	1			1		
≥12	1.39	(0.76–2.42)	0.33	1.52	(0.85–2.76)	0.21
Second surgery before treatment						
No	1					
Yes	0.73	(0.42–1.43)	0.51			
Maximum tumor diameter (mm)	0.61	(0.37–1.18)	0.19			

**Table 3:** Univariate and multivariate analysis of prognostic factors for overall survival in 176 patients with recurrent glioblastoma multiforme.

	Univariate effect			Multivariate effect		
	HR	95% CI	*P*	HR	95% CI	*P*
Gender						
Female	1			1		
Male	0.97	(0.71–1.56)	0.91	1.41	(0.80–1.74)	0.25
Age (years)	0.90	(0.72–1.26)	0.79	0.85	(0.65–1.31)	0.61
KPS score, *n* (%)						
<70	1			1		
≥70	0.63	(0.45–0.89)	0.01	0.58	(0.39–0.76)	0.01
Steroid use						
No	1					
Yes	0.90	(0.71–1.27)	0.85	0.81	(0.62–1.27)	0.63
Type of first surgery, *n* (%)						
Biopsy/partial resection	1			1		
Subtotal/total resection	0.76	(0.38–1.23)	0.51	0.82	(0.49–1.38)	0.74
MGMT status						
Unmethylated	1			1		
Methylated	0.95	(0.67–1.35)	0.82	0.88	(0.58–1.26)	0.71
Time from original diagnosis (months)						
<12	1			1		
≥12	1.54	(0.64–2.09)	0.46	1.41	(0.69–1.99)	0.53
Second surgery before treatment						
No	1					
Yes	0.87	(0.57–1.31)	0.90			
Maximum tumor diameter (mm)	0.73	(0.45–1.39)	0.47			

**Table 4:** Summary of adverse events.

Adverse event	Grade: number of patients			
	1	2	3	4
Nonhematologic toxicity				
Fatigue	55	16	5	—
Proteinuria	23	28	—	—
Hypertension	8	13	4	3
Hypophonia	12	—	—	—
Hyperpigmentation	5	—	—	—
Hemorrhage, CNS	5	2	—	—
Hemorrhage, GI	—	2	—	—
Epistaxis	4	—	—	—
Stroke	1	1	1	—
DVT	—	2	3	—
PE	3	1	1	—
Wound dehiscence	4	1	1	—
Infection	2	—	—	—
Rash	—	—	—	—
GI perforation/fistula	—	—	—	—
Transaminase elevation	—	—	—	—
Hematologic toxicity	—	—	—	—
Anemia	4	2	2	—
Neutropenia	5	6	18	3
Thrombocytopenia	9	17	14	2

## References

[1] Louis D. N., Ohgaki H., Wiestler O. D. (2007). The 2007 WHO classification of tumours of the central nervous system. *Acta Neuropathologica*.

[2] Oszvald Á., Güresir E., Setzer M. (2012). Glioblastoma therapy in the elderly and the importance of the extent of resection regardless of age: clinical article. *Journal of Neurosurgery*.

[3] Stupp R., Mason W. P., van den Bent M. J. (2005). Radiotherapy plus concomitant and adjuvant temozolomide for glioblastoma. *The New England Journal of Medicine*.

[4] Stupp R., Hegi M. E., Mason W. P. (2009). Effects of radiotherapy with concomitant and adjuvant temozolomide versus radiotherapy alone on survival in glioblastoma in a randomised phase III study: 5-year analysis of the EORTC-NCIC trial. *The Lancet Oncology*.

[5] Friedman H. S., Prados M. D., Wen P. Y. (2009). Bevacizumab alone and in combination with irinotecan in recurrent glioblastoma. *Journal of Clinical Oncology*.

[6] Kreisl T. N., Kim L., Moore K. (2009). Phase II trial of single-agent bevacizumab followed by bevacizumab plus irinotecan at tumor progression in recurrent glioblastoma. *Journal of Clinical Oncology*.

[7] Chamberlain M. C., Johnston S. K. (2010). Salvage therapy with single agent bevacizumab for recurrent glioblastoma. *Journal of Neuro-Oncology*.

[8] de Rossi A., Rossi L., Laudisi A. (2006). Focus on fotemustine. *Journal of Experimental & Clinical Cancer Research*.

[9] Avril M. F., Aamdal S., Grob J. J. (2004). Fotemustine compared with dacarbazine in patients with disseminated malignant melanoma: a phase III study. *Journal of Clinical Oncology*.

[10] Vaccaro V., Fabi A., Vidiri A. (2014). Activity and safety of bevacizumab plus fotemustine for recurrent malignant gliomas. *BioMed Research International*.

[11] Yamamoto D., Iwase S., Tsubota Y. (2012). Bevacizumab in the treatment of five patients with breast cancer and brain metastases: Japan breast cancer research network-07 trial. *OncoTargets and Therapy*.

[12] Arakawa Y., Mizowaki T., Murata D. (2013). Retrospective analysis of bevacizumab in combination with ifosfamide, carboplatin, and etoposide in patients with second recurrence of glioblastoma. *Neurologia Medico-Chirurgica*.

[13] Tabouret E., Barrie M., Thiebaut A. (2013). Limited impact of prognostic factors in patients with recurrent glioblastoma multiforme treated with a bevacizumab-based regimen. *Journal of Neuro-Oncology*.

[14] Vredenburgh J. J., Desjardins A., Herndon J. E. (2007). Bevacizumab plus irinotecan in recurrent glioblastoma multiforme. *Journal of Clinical Oncology*.

[15] Soffietti R., Trevisan E., Bertero L. (2014). Bevacizumab and fotemustine for recurrent glioblastoma: a phase II study of AINO (Italian Association of Neuro-Oncology). *Journal of Neuro-Oncology*.

[16] Poulsen H. S., Grunnet K., Sorensen M. (2009). Bevacizumab plus irinotecan in the treatment patients with progressive recurrent malignant brain tumours. *Acta Oncologica*.

[17] Reardon D. A., Desjardins A., Vredenburgh J. J. (2009). Metronomic chemotherapy with daily, oral etoposide plus bevacizumab for recurrent malignant glioma: a Phase II Study. *British Journal of Cancer*.

[18] Verhoeff J. J. C., Lavini C., van Linde M. E. (2010). Bevacizumab and dose-intense temozolomide in recurrent high-grade glioma. *Annals of Oncology*.

[19] Reardon D. A., Desjardins A., Peters K. B. (2011). Phase 2 study of carboplatin, irinotecan, and bevacizumab for recurrent glioblastoma after progression on bevacizumab therapy. *Cancer*.

[20] Hasselbalch B., Lassen U., Hansen S. (2010). Cetuximab, bevacizumab, and irinotecan for patients with primary glioblastoma and progression after radiation therapy and temozolomide: a phase II trial. *Neuro-Oncology*.

[21] Sathornsumetee S., Desjardins A., Vredenburgh J. J. (2010). Phase II trial of bevacizumab and erlotinib in patients with recurrent malignant glioma. *Neuro-Oncology*.

[22] Chinot O. L., Wick W., Mason W. (2014). Bevacizumab plus radiotherapy-temozolomide for newly diagnosed glioblastoma. *The New England Journal of Medicine*.

[23] Lacroix M., Abi-Said D., Fourney D. R. (2001). A multivariate analysis of 416 patients with glioblastoma multiforme: prognosis, extent of resection, and survival. *Journal of Neurosurgery*.

[24] Filippini G., Falcone C., Boiardi A. (2008). Prognostic factors for survival in 676 consecutive patients with newly diagnosed primary glioblastoma. *Neuro-Oncology*.

[25] Stark A. M., Stepper W., Mehdorn H. M. (2010). Outcome evaluation in glioblastoma patients using different ranking scores: KPS, GOS, mRS and MRC. *European Journal of Cancer Care*.

[26] Metellus P., Coulibaly B., Nanni I. (2009). Prognostic impact of O6-methylguanine-DNA methyltransferase silencing in patients with recurrent glioblastoma multiforme who undergo surgery and carmustine wafer implantation: a prospective patient cohort. *Cancer*.

[27] Felsberg J., Thon N., Eigenbrod S. (2011). Promoter methylation and expression of MGMT and the DNA mismatch repair genes MLH1, MSH2, MSH6 and PMS2 in paired primary and recurrent glioblastomas. *International Journal of Cancer*.

[28] Norden A. D., Lesser G. J., Drappatz J. (2013). Phase 2 study of dose-intense temozolomide in recurrent glioblastoma. *Neuro-Oncology*.

[29] Brandes A. A., Tosoni A., Cavallo G. (2006). Temozolomide 3 weeks on and 1 week off as first-line therapy for recurrent glioblastoma: phase II study from Gruppo Italiano Cooperativo di Neuro-Oncologia (GICNO). *British Journal of Cancer*.

[30] Wick A., Felsberg J., Steinbach J. P. (2007). Efficacy and tolerability of temozolomide in an alternating weekly regimen in patients with recurrent glioma. *Journal of Clinical Oncology*.

[31] Sadones J., Michotte A., Veld P. (2009). MGMT promoter hypermethylation correlates with a survival benefit from temozolomide in patients with recurrent anaplastic astrocytoma but not glioblastoma. *European Journal of Cancer*.

[32] Zhang K., Wang X.-Q., Zhou B., Zhang L. (2013). The prognostic value of MGMT promoter methylation in Glioblastoma multiforme: a meta-analysis. *Familial Cancer*.

[33] Hervey-Jumper S. L., Berger M. S. (2014). Reoperation for recurrent high-grade glioma: a current perspective of the literature. *Neurosurgery*.

[34] Lai A., Tran A., Nghiemphu P. L. (2011). Phase II study of bevacizumab plus temozolomide during and after radiation therapy for patients with newly diagnosed glioblastoma multiforme. *Journal of Clinical Oncology*.

[35] Ahmadloo N., Kani A.-A., Mohammadianpanah M. (2013). Treatment outcome and prognostic factors of adult glioblastoma multiforme. *Journal of the Egyptian National Cancer Institute*.

